# Elastic K-means using posterior probability

**DOI:** 10.1371/journal.pone.0188252

**Published:** 2017-12-14

**Authors:** Aihua Zheng, Bo Jiang, Yan Li, Xuehan Zhang, Chris Ding

**Affiliations:** 1 Anhui University, Hefei, China; 2 Anhui Broadcasting Movie and Television College, Hefei, China; Northwestern Polytechnical University, UNITED STATES

## Abstract

The widely used K-means clustering is a hard clustering algorithm. Here we propose a Elastic K-means clustering model (EKM) using posterior probability with soft capability where each data point can belong to multiple clusters fractionally and show the benefit of proposed Elastic K-means. Furthermore, in many applications, besides vector attributes information, pairwise relations (graph information) are also available. Thus we integrate EKM with Normalized Cut graph clustering into a single clustering formulation. Finally, we provide several useful matrix inequalities which are useful for matrix formulations of learning models. Based on these results, we prove the correctness and the convergence of EKM algorithms. Experimental results on six benchmark datasets demonstrate the effectiveness of proposed EKM and its integrated model.

## Introduction

Data clustering, a method of unsupervised learning and a common technique for statistical data analysis has currently been widely used in machine learning [1, 2], pattern recognition [3, 4], image analysis [5, 6] and bioinformatics [7–9]. As one of the most popular clustering methods, K-means has drawn lots of attention and been widely used for data clustering. Recent work has revealed that K-means can be represented by matrix factorization formulation, and thus can deduce many kind of K-means variants [10–15]. From the point of view of determinacy, K-means clustering approaches are hard clustering where a data point exactly belongs to one particular class. However, in many cases, this is not realistic since not all the data point distinctly belongs to one single class especially for the outliers which may have equivalent similarities to every/some classes.

On the other hand, above K-means inspired methods generally deal with the attribute information (feature vectors) of data. In real world applications, besides attribute information, we have various pairwise relations between data points which are expressed as **graph** data. There exist many clustering methods that utilize graph data, such as Ratio Cut based methods [16, 17] and Normalized Cut based methods [18, 19] and Min and Max Cut based methods [20, 21]. In essence, these clustering methods first embed graph nodes in low-dimensional space using linear embedding method PCA and nonlinear method IsoMAP [22–24], Local linear Embedding (LLE) [25–27], Local Tangent Space Alignment [26, 28, 29] etc, where feature vector information is utilized to obtain the final clustering results. However, It’s one-sided to just take into account of one single information.

Inspired by recent works on matrix factorization based K-means models, In this paper, we first propose a **Elastic** clustering model using posterior probability (named as Elastic K-means, EKM). The main observation of our Elastic K-means model is that the clustering indicator from K-means can be interpreted as the posterior cluster probabilities during the factorization procedure. The key point of the Elastic K-means is that each data point is assigned to the clusters scattered into several possible classes according to its posterior probabilities. Then we extend our EKM to graph EKM (gEKM), which simultaneously utilizes both feature vectors and pairwise relations between data points. Specifically, gEKM integrates the proposed Elastic K-means with Normalized Cut clustering. In order to evaluate the effectiveness of proposed EKM and gEKM, we implement them on six benchmark datasets. The promising experimental results demonstrate the benefit of the proposed Elastic K-means models.

## Elastic K-means and related works

### K-means

Given data matrix *X* = (*x*_1_, ⋯, *x*_*n*_) ∈ ℜ^*p*×*n*^ denoting *n* data points. The objective of K-means clustering is to find the cluster centroids *c*_*k*_, *k* = 1, ⋯ , *K* by minimizing the following cost function:

$$\begin{array}{c} J_{km}=\sum_{i=1}^{n}\sum_{k=1}^{K}{∥x_{i}-c_{k}∥}^{2} \end{array}$$ (1)

Our work starts with the observation that the above K-means clustering objective can be reformulated as below:

**Proposition 1**. *The objective function* Eq (1) *can be rewritten as*:

$$\begin{array}{l} \begin{array}{cc} \underset{G}{\text{min}} & J={∥X-XGG^{T}∥}^{2} \end{array}\\ s.t.\;G^{T}G=N^{-1},\;\;G_{ik}\in \left\{0,1/\sqrt{n_{k}}\right\}. \end{array}$$ (2)

$N=\text{diag}(n_{1},\cdots ,n_{k})$ where *n*_*i*_, (*i* = 1, ⋯ , *k*) denotes the number of data points in each cluster. Note that ∥*A*∥ denotes the Frobenius norm of a matrix A. And *G* ∈ ℜ^*n*×*K*^ is the *normalized* cluster indicators, i.e.,

$$\begin{array}{c} G_{i,k}=\{\begin{array}{cc} 1/\sqrt{n_{k}} & \text{if}\;x_{i}\in c_{k},\\ 0 & \text{otherwise}. \end{array} \end{array}$$ (3)

**Proof of Proposition 1**.

Let us introduce the standard cluster indicator

$$\begin{array}{c} {\tilde{G}}_{i,k}=\{\begin{array}{cc} 1 & \text{if}\;x_{i}\in c_{k},\\ 0 & \text{otherwise}. \end{array} \end{array}$$ (4)

Then, Eq (1) can be equivalently written as:

$$\begin{array}{l} \begin{array}{cc} \underset{C,\tilde{G}}{\text{min}} & J_{km}={∥X-C{\tilde{G}}^{T}∥}^{2} \end{array}\\ s.t.\;\;{\tilde{G}}^{T}\tilde{G}=I,\;\;{\tilde{G}}_{ik}\in \left\{0,1\right\} \end{array}$$ (5)

where *C* = (*c*_1_, *c*_2_, ⋯ , *c*_*K*_) ∈ ℜ^*p*×*K*^ are cluster centroids. The orthogonality of *G* is preserved in a relaxation of class indicator matrix [21]. Now, the cluster centroids can be written as $c_{k}=X{\tilde{g}}_{k}/n_{k}$, where ${\tilde{g}}_{k}$ is the *k*-th column of $\tilde{G}$, or equivalently, $C=\left(c_{1},\cdots ,c_{K}\right)=X\tilde{G}N^{-1}$. Thus:

$${∥X-C{\tilde{G}}^{T}∥}^{2}={∥X-X\tilde{G}N^{-1}{\tilde{G}}^{T}∥}^{2}={∥X-XGG^{T}∥}^{2}$$ (6)

where $G=\tilde{G}N^{-1/2}$ absorbs the unknown N.

### Elastic K-means

**Proposition 1** provides an natural way to form the Elastic K-means. First, the orthogonal constraint *G*^*T*^ *G* = *I* ensures that one data point belongs to only one cluster as in [21, 30]. We **relax** this constraint, so that each data point can belong to multiple clusters. Because the formulation Eq (2) is a matrix decomposition form, the constraint *G*^*T*^ *G* = *I* will be satisfied approximately.

Second, since the mixed signs of *G* may deviate the results severely from the true solution of clustering [21, 30], we relax the discrete constraint $G_{ik}\in \left\{0,1/\sqrt{n_{k}}\right\}$, to a simple nonnegativity constraint *G* ≥ 0. Thus, the final model is formulated as:

$$\begin{array}{l} \begin{array}{cc} \underset{G}{\text{min}} & J_{ekm}={∥X-XGG^{T}∥}^{2} \end{array}\\ s.t.\;\;G\geq 0 \end{array}$$ (7)

Note that the input data *X* has mixed signs.

We call this Elastic K-means (EKM). (1) This model is invariant w.r.t. *X* → *βX*, *β* ∈ ℜ is any constant. This is the same as the standard K-means. (2) Similar to K-means, the expected clustering centroids are adopted during the updating iterations. (3) different from the traditional K-means, the elastic indicator (interpreted as the posterior probability which is a fraction) performs a smooth convergency during updating. In fact, all the NMF-based methods we discussed above have K-means clustering interpretations when the factor G is orthogonal (*G*^*T*^ *G* = *I*), which in turn means they can be regarded as the relaxations of K-means.

### Related works

Our goal is to study the case where a data point is not restricted to belong to a single cluster. Three related clustering models are (A) Gaussian mixtures [31, 32], (B) Fuzzy C-means [33–35], (C) Fuzzy K-means [12, 14, 36, 37]. In general, Gaussian mixtures works only for low-dimensional data, typically *d* ≤ 10, therefore they are not suitable for high dimensional data. Fuzzy C-means solves

$$\begin{array}{c} \underset{u,c_{k}}{\text{min}}\;\;\;J_{fcm}=\sum_{i=1}^{n}\sum_{k=1}^{K}u_{ik}^{m}{∥x_{i}-c_{k}∥}^{2} \end{array}$$ (8)

where *u*_*ik*_ is the membership of data point *x*_*i*_ belonging to cluster *k* and ∑_*k*_ *u*_*ik*_ = 1.

Our model differs from Fuzzy C-means on two aspects: (1) Cluster distribution of single data point *x*_*i*_ is implicit in our model, whereas it is explicit in Fuzzy C-means. (2) The requirement that the posterior probability ∑_*k*_ *u*_*ik*_ = 1 for any data point *x*_*i*_, but the actual distribution used in Eq (8) is $u_{ik}^{m}$, *m* = 2 in most cases. Thus the normalization of the actual cluster distribution is $z_{i}=\sum_{k}u_{ik}^{m}<1$ and *z*_*i*_ is different for different data point *x*_*i*_. This theoretical/conceptual drawback for Fuzzy C-means is not present in our Elastic K-means. Fuzzy K-means solves

$$\begin{array}{c} \underset{w,c_{k}}{\text{min}}\;\;\;J_{fkm}=\sum_{i=1}^{n}\sum_{k=1}^{K}w_{ik}{∥x_{i}-c_{k}∥}^{2} \end{array}$$ (9)

where *w*_*ik*_ is the membership of data point *x*_*i*_ belonging to cluster *k* and ∑_*k*_ *w*_*ik*_ = 1. The main difference between Fuzzy K-means and our method is, the soft/fuzzy capability is achieved by exploring the corresponding weight *w*_*ik*_ (or *u*_*ik*_ in Fuzzy C-means) for each data point *x*_*i*_, while our Elastic K-means achieves this via the soft/fuzzy cluster indicator *G*, which is more explicit. Note that, the concept of soft/fuzzy K-means (or C-means)has been mentioned in a group of literatures [38–42], which also have the fuzzy/soft capability for data clustering. However, these soft clustering methods are generally derived from Fuzzy C-means or Fuzzy K-means and their variants, which are essentially different from the proposed Elastic K-means model.

Because in our model, *G* is nonnegative. In some sense, our model also relates to NMF. There exists a very broad category of work along NMF direction. We refer to a recent survey [43].

## Computational algorithm and analysis

### Algorithm

An effective updating algorithm can be derived to solve Elastic K-means problem. The algorithm iteratively updates the current solution as follows:

$$\begin{array}{c} G_{ik}\leftarrow G_{ik}\sqrt[{4}]{\frac{{\left(2AG+BGG^{T}G+GG^{T}BG\right)}_{ik}}{{\left(2BG+AGG^{T}G+GG^{T}AG\right)}_{ik}}} \end{array}$$ (10)

The pseudo codes of EKM is illustrated as **Algorithm 1**.

**Algorithm 1**: Elastic K-means Algorithm

 **Input**: data *X*

**1** (1) Initialize *G*^0^.

**2** Construct the indicator *G*: *G*_*ik*_ = 1 if *x*_*i*_ belongs to cluster *k*. otherwise, *G*_*ik*_ = 0

**3** (2) Update *G*

**4** **while not converged**

**5** $G_{ik}\leftarrow G_{ik}\cdot \sqrt[{4}]{\frac{{\left(2AG+BGG^{T}G+GG^{T}BG\right)}_{ik}}{{\left(2BG+AGG^{T}G+GG^{T}AG\right)}_{ik}}}$

**6** where

**7** *A* = (|(*X*^*T*^ *X*)_*ik*_| + (*X*^*T*^ *X*)_*ik*_)/2

**8** *B* = (|(*X*^*T*^ *X*)_*ik*_| − (*X*^*T*^ *X*)_*ik*_)/2

**9** end

The convergence of the proposed algorithm can be found in the supplementary.

### Updating algorithms for quartic models

There are many matrix models involving matrix variables to the 4th power. The simplest is:

$$\begin{array}{c} \underset{G}{\text{min}}\;{∥W-GG^{T}∥}_{F}^{2} \end{array}$$ (11)

where *W* = *W*^*T*^ is symmetric. A number of papers claim that the updating algorithm for this model is

$$\begin{array}{c} G_{ik}\leftarrow G_{ik}\frac{{\left(WG\right)}_{ik}}{{\left(GG^{T}G\right)}_{ik}} \end{array}$$ (12)

In fact, this updating algorithm does not guarrentee the monotonic decreasy of the objective function value. Using the inequality of quartic matrix of Eq (S15) in the supplementary, we can easily prove that the following updating algorithm;

$$\begin{array}{c} G_{ik}\leftarrow G_{ik}\sqrt[{4}]{\frac{{\left(WG\right)}_{ik}}{{\left(GG^{T}G\right)}_{ik}}} \end{array}$$ (13)

which guarrentees decrease of objective, and thus the convergence.

## Benefit of Elastic K-means

**Fig 1:**
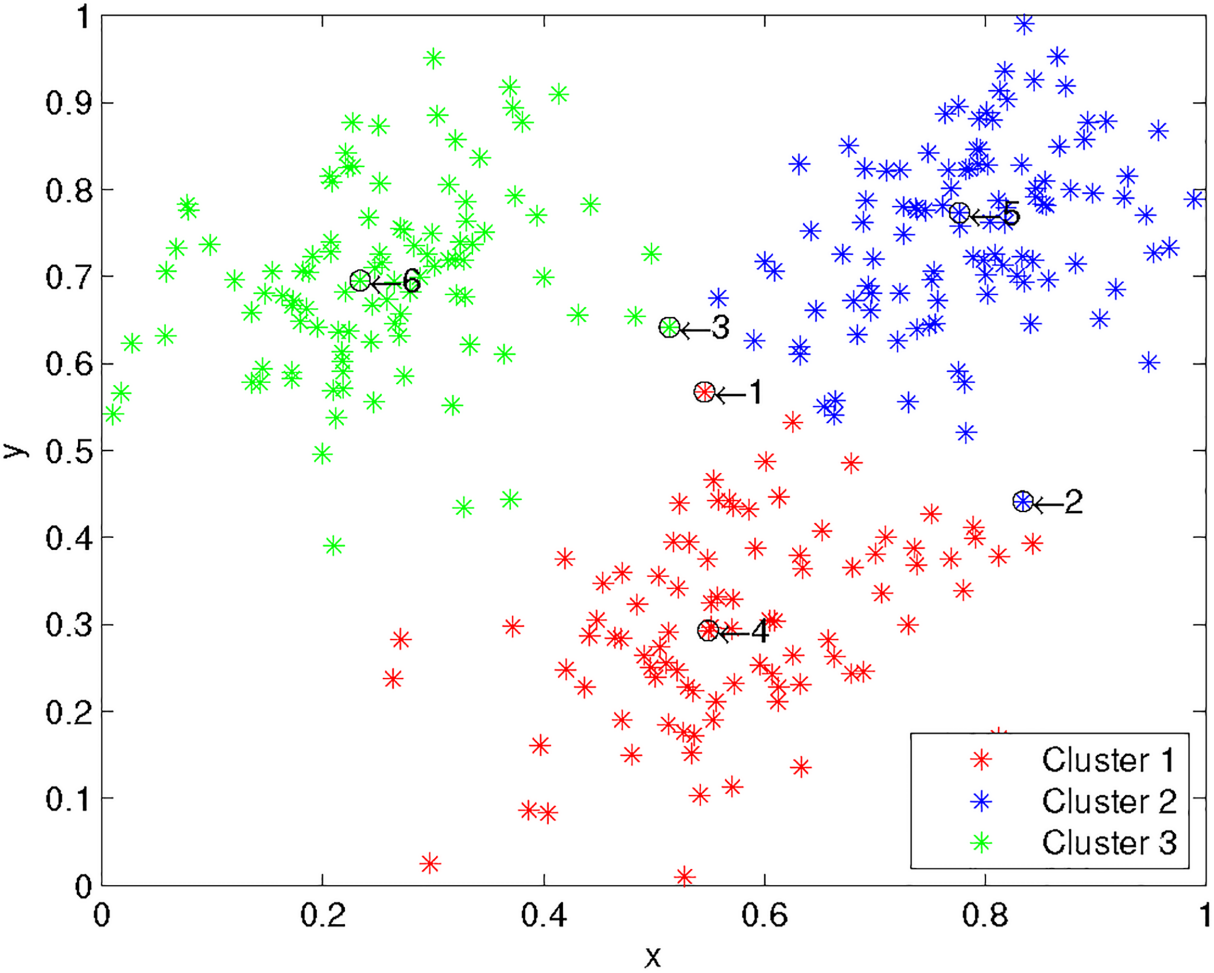
EKM clustering results on 2 dimensional 3 Gaussian clusters. x and y axes denote the first and the second dimension respectively.

One main drawback of standard K-means is that each data point is assigned to a single cluster (hard clustering), but in real data, many data points are somewhere in-between different clusters centers (such as points 1, 2, 3 shown in Fig 1). One clear benefit of the proposed Elastic K-means is that these ambiguous data points are assigned into several nearby clusters, i.e., their posterior probabilities of cluster assignment are nearly evenly distributed.

**Fig 2:**
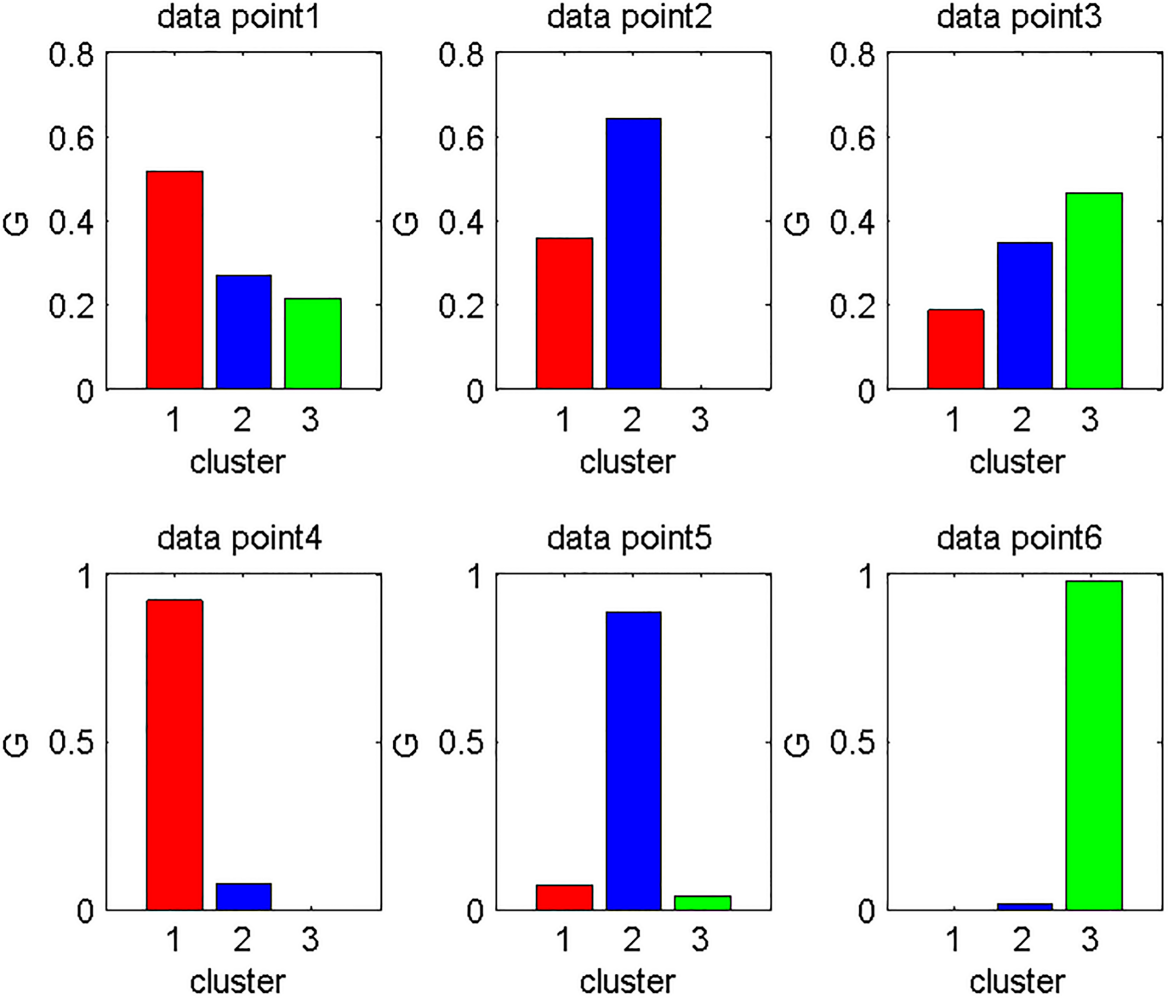
An example of the posterior G of 3 ambiguous data points and 3 sharp data points on above EKM clustering results.

Fig 1 shows the EKM results on one 2D dataset of 3 randomly generated Gaussian clusters. The EKM cluster results are indicated as red, green and blue stars respectively. Point 4, 5, 6 clearly belong to their assigned clusters. This fact is correctly encoded in the EKM model, as shown in the posterior probabilities in Fig 2: they are sharply concentrated on a single cluster.

On the other hand, points 1, 2, 3 are ambiguous: they are in-between different cluster centers. A good ‘elastic’ clustering model should assign these points to nearby clusters with nearly-equal posterior probabilities. Indeed, this desirable fact is correctly encoded in the EKM model, as shown in the posterior probabilities in Fig 2: they are nearly evenly distributed on nearby clusters.

**Posterior Probability**. In this and following sections, we normalize *G* to

$$\begin{array}{c} G_{ik}\leftarrow G_{ik}/\sum_{k}G_{ik} \end{array}$$ (14)

so that ∑_*k*_ *G*_*ik*_ = 1 for data point *x*_*i*_.

A natural question is: how to identify those ambiguous data points such points 1, 2, 3 in Fig 2. For this purpose, we need to see how the posterior probability

$$\begin{array}{c} p_{i}=G_{i:}=\left(G_{i1},G_{i2},\cdots ,G_{ik}\right) \end{array}$$ (15)

for data point *x*_*i*_ is distributed.

We use a useful quality to indicate approximately how sharply the distribution is peaked around the highest cluster. This is the gap in the posterior distribution: the difference between the highest and second highest peaks:

$$\begin{array}{c} \Delta_{i}=\left(G_{ik_{1}}-G_{ik_{2}}\right)/G_{ik_{1}} \end{array}$$ (16)

where

$$\begin{array}{c} k_{1}=\arg\max_{1\leq k\leq K}G_{ik},k_{2}=\arg\max_{1\leq k\leq K,j\neq k_{1}}G_{ik} \end{array}$$ (17)

The idea is simple. (A) If a data point is well inside a cluster, such as points 4, 5, 6 in Fig 2, the highest peak is large and the second highest peak is small. Thus the gap is big. (B) If a data point is in-between different clusters, such as points 1, 2, 3, the difference between the highest peak and the second highest peak is small. Thus the gap is small. From these situations, the gap is useful indication of how sharply the posterior probability is distributed.

**Fig 3:**
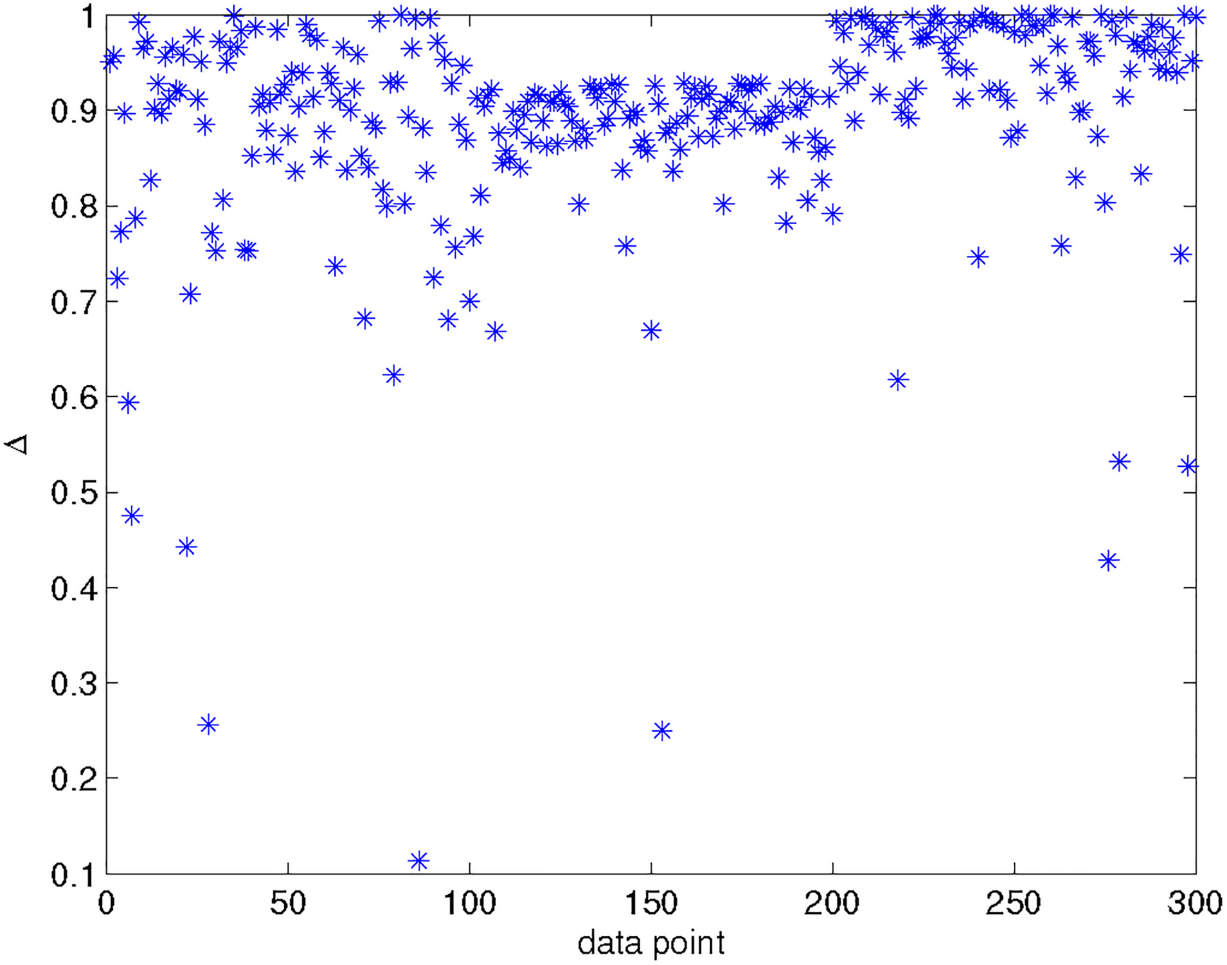
The gap in the posterior distribution: The difference between the highest and second highest peaks.

Fig 3 shows the distribution of the gap Δ_*i*_ of the 300 data points shown in Fig 1. Noted that, the lager Δ_*i*_, the sharper that the data point belongs to a single class. Obviously, there are many ambiguous data points which can be easily detected using the gap. These studies indicate that Elastic K-means is able to detect the fuzzy/soft characteristics of data clustering.

## Integrating Elastic K-means and normalized cut

EKM uses only vector (sometimes called attribute) data *X*. In many applications, the input data consists of both vector data *X* and similarity data *W* (also called graph data since *W*_*ij*_ represents the similarity between data points *i* and *j*). For this situation, we can easily extend EKM to utilize the similarity data *W* for more effective elastic clustering.

We incorporate the similarity data through the Normalized Cut [18, 19] formalism. This extension form a single clustering formulation for input data with both vector and similarity data.

The Ncut (Normalized Cut) is defined as:

$$\begin{array}{c} \max_{Q}\;Tr\left(Q^{T}D^{-\frac{1}{2}}WD^{-\frac{1}{2}}Q\right)\\ s.t.\;Q^{T}Q=I,Q\in \left\{0,1\right\} \end{array}$$ (18)

where *D* = *diag*(*d*_1_, ⋯ , *d*_*n*_), *d*_*i*_ = ∑_*j*_ *W*_*ij*_.

**Proposition 2**. Optimization problem Eq (18) is equivalent to

$$\begin{array}{cc} \underset{Q}{\text{min}} & \;∥S-QQ^{T}{∥}^{2}\\ s.t. & Q^{T}Q=I,Q\in \left\{0,1\right\}. \end{array}$$ (19)

with the same constraints of Eq (18), where $S=D^{-\frac{1}{2}}WD^{-\frac{1}{2}}$

**Proof**. Because ∥*S* − *QQ*^*T*^∥^2^ = *Tr*(*S*^2^ − 2*Q*^*T*^ *SQ* + *QQ*^*T*^ *QQ*^*T*^), the first term and the last term are both constant due to *Q*^*T*^ *Q* = *I*. The minimization of −2*Q*^*T*^ *SQ* is the same as Eq (18).

In the same manner as EKM in Eq (7), we relax the orthogonal constraint *Q*^*T*^ *Q* = *I* to be nonnegative, then Eq (19) can be rewritten as:

$$\begin{array}{cc} \underset{Q}{\text{min}} & \;∥S-QQ^{T}{∥}^{2}\\ s.t. & Q\geq 0 \end{array}$$ (20)

Since *Q* is also the cluster indicators as *G* in EKM, we can integrate EKM and NCut by simplifying/approximating these constraints into *G* ≥ 0. The above model is now expressed in a simplified way:

$$\begin{array}{c} \underset{G}{\text{min}}\;2{∥X-XGG^{T}∥}^{2}+\alpha ∥S-GG^{T}{∥}^{2}\\ s.t.\;G\geq 0 \end{array}$$ (21)

We call this model as graph EKM (simplified as gEKM in the following parts) since Ncut is a popular approach for graph data clustering. It naturally incorporates both the feature/attribute data *X* and pairwise relations *W*.

### Analysis

Because the model is invariant with *X* → *βX*, let *β* = 1/∥*X*∥, which implies the input *X* is normalized: ${∥X∥}^{2}=\Sigma_{ij}X_{ij}^{2}=1$.

The above objective can be written as:

$$\begin{array}{c} J=Tr[2X^{T}X+\alpha \left(S^{2}-I\right)-2G^{T}\left(X^{T}X+\alpha S\right)G] \end{array}$$ (22)

The first 2 terms are independent of *G*. Thus the above optimization becomes

$$\begin{array}{c} \underset{G}{\text{min}}\;Tr\;G^{T}\left(X^{T}X+\alpha S\right)G\\ s.t.\;G\geq 0 \end{array}$$ (23)

where *X* is normalized.

This expression reveals an important **insight** that the gEKM model is a discrete combinatorial optimization.

### Algorithm 2

The optimization problem of Eq (21) can be solved by the following iterative algorithm:

$$\begin{array}{c} G_{ik}\leftarrow G_{ik}\sqrt[{4}]{\frac{{\left(2AG+BGG^{T}G+GG^{T}BG+2\alpha_{1}SG\right)}_{ik}}{{\left(2BG+AGG^{T}G+GG^{T}AG+2\alpha_{1}GG^{T}G\right)}_{ik}}} \end{array}$$ (24)

where *α*_1_ = *α* * ∥*X*∥^2^, and *A*, *B* are defined in **Algorithm 1**. The proof of convergence can be similarly established as **Algorithm 1**.

## Experiments

We perform experiments on following six benchmark datasets described as Table 1.

**Table 1:** Dataset names, number of data samples, data dimension and number of classes of the 6 benchmark datasets.

Dataset	#Sample	#Dimension	#Class
AT&T	400	1024	40
USPS	9298	256	10
MNIST	1000	784	10
COIL20	1440	1024	20
Isolet1	1560	617	26
BinAlph	1014	320	26

**AT & T** (http://www.cl.cam.ac.uk/research/dtg/attarchive/facedatabase.html) face dataset contains ten different images of each 40 distinct subjects. For some subjects, the images were taken at different times, varying the lighting, facial expression and facial details (glass/no glass). All images were taken against a dark homogeneous background with the subjects in an upright, frontal, position. We stretch each image into a vector.

**USPS** (http://www.cs.nyu.edu/roweis/data.html) is a handwritten digit (1–10) database and we select 1000 images (100 images for every digit).

**MNIST** (http://www.cs.nyu.edu/roweis/data.html) is a handwritten digit database. Each image is centered (according to the center of mass of the pixel intensities) on a 2828 grid. In our experiments, we randomly choose 1000 images (i.e., each digit has 100 images). We reshape each image into one vector.

**COIL20** (http://www.cs.columbia.edu/CAVE/software/softlib/coil-20.php) contains 20 objects. Each image of the same object is taken 5 degrees apart as the object is rotated on a turntable and each object has 72 images. The size of each image is 3232 pixels, with 256 grey levels per pixel. Each image is represented by a 1024 dimensional vector.

**Isolet1** (http://archive.ics.uci.edu/ml/datasets/ISOLET) dataset was generated by 150 subjects spoken the name of each letter of the alphabet twice. The speakers are grouped into sets of 30 speakers each, and are referred to as isolet1, isolet2, isolet3, isolet4, isolet5. The features include spectral coefficients, contour features, sonorant features, pre-sonorant features, and post-sonorant features. In our experiment, we utilize subset isolet1 only.

**BinAlpha** (http://www.cs.nyu.edu/roweis/data.html) contains 26 binary hand-written alphabets and we select 30 images for every alphabet. We stretch each image into one vector.

Clustering accuracy is used to measure the performances. Once the clustering solution is computed, the confusion matrix is calculated using the Hungarian algorithm [44] is employed to match obtained clusters with the ground truth classes. The accuracy of this matching is computed as the clustering accuracy.

In all experiments, for each dataset, we first run 20 K-means clustering with random starts. We pick the best (lowest clustering objective function value) result of these 20 runs, record the corresponding clustering accuracy as the result of K-means.

Starting from this K-means result, we run NMF [11], FCM [34], MinMaxCut [21], EKM, gEKM, and record the corresponding results. For Ncut [18], we run K-means in the eigenspace 20 times with random starts, and pick the best (lowest K-means clustering objective function value) result of these 20 runs.

### Choice of parameter *α*

**Fig 4:**
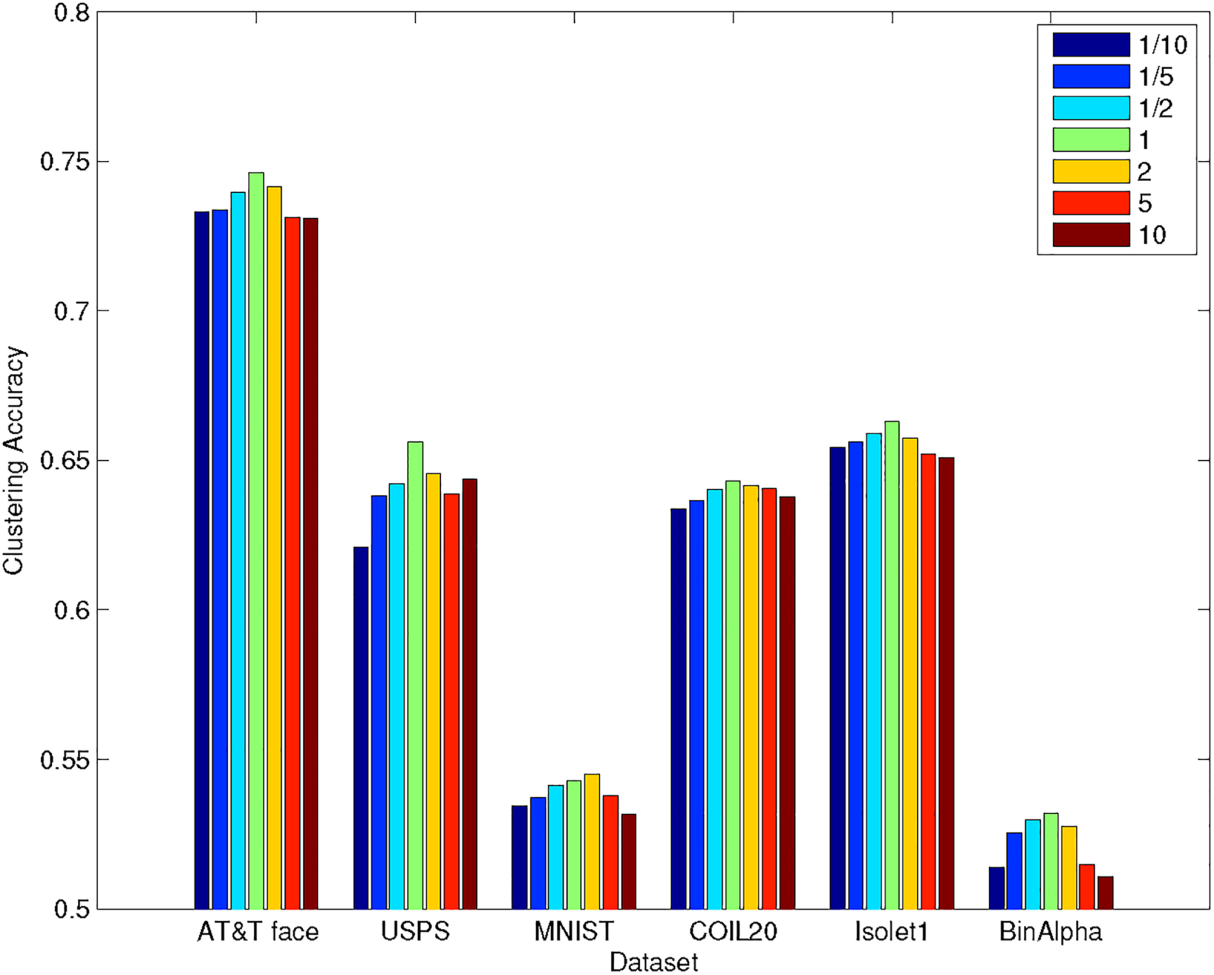
Clustering accuracy for gEKM against *α* on six datasets in full dimension.

The clustering accuracy of gEKM depends on parameter *α*. If *α* > 1, the similarity part (2nd term of Eq (21)) is more important. If *α* < 1, the vector attribute part (first term of Eq (21)) is more important. For this reason, the proper range of choice for *α* is [1/10, 1/5, 1/2, 1, 2, 5, 10]. We show the clustering results against *α* on all six datasets in Fig 4 (here *X* is in full dimension).

We observe that (1) The results are not very sensitive to *α*. (2) *α* = 1 generally gives best results, which in turn means the vector attribute and the similarity part have more or less the balanced contribution. From these observations, we fix *α* to 1 in the following experiments.

### Convergency visualization

**Fig 5:**
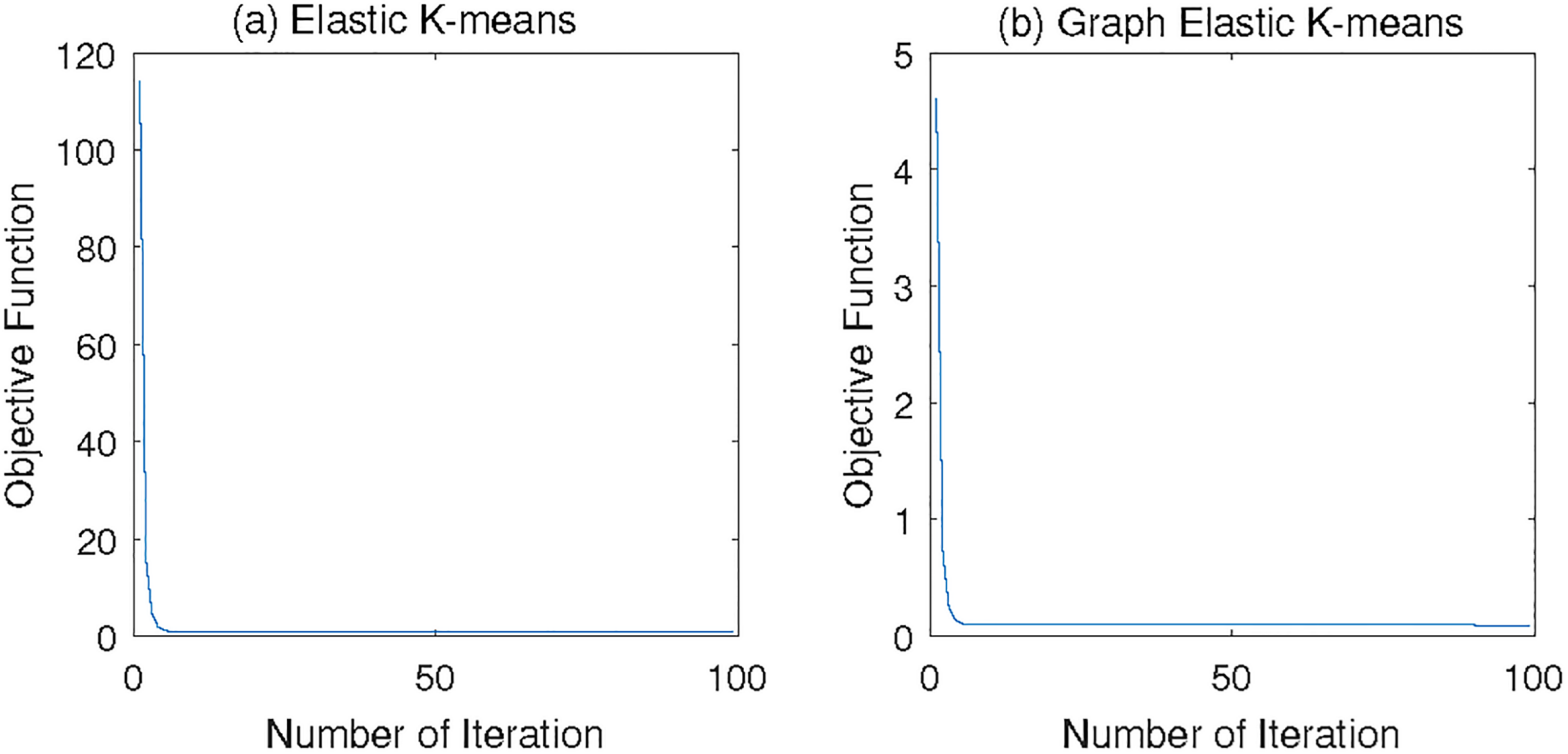
Changes of the objective function of the EKM and gEKM in 100 iterations.

Fig 5 visually illustrates the changes of the objective functions of Eqs (7) and (21), which demonstrates the convergency of the objective functions.

### Results on clustering

We compare our methods, EKM and gEKM to K-means, NMF [11], FCM [34], Ncut [18] and MixMaxCut [21] on the six datasets. For EKM, Ncut [18] and MinMaxCut [21], we compute the graph similarity *W* as:

$$\begin{array}{c} W_{ij}=\exp(-∥x_{i}-x_{j}{∥}^{2}/cd^{2}) \end{array}$$ (25)

where *c* = 0.7 and *d* is the average distances between each *x*_*i*_ and its 7 nearest neighbors. We set *W*_*ii*_ = 0 and construct $S=D^{-\frac{1}{2}}WD^{-\frac{1}{2}}$.

**Fig 6:**
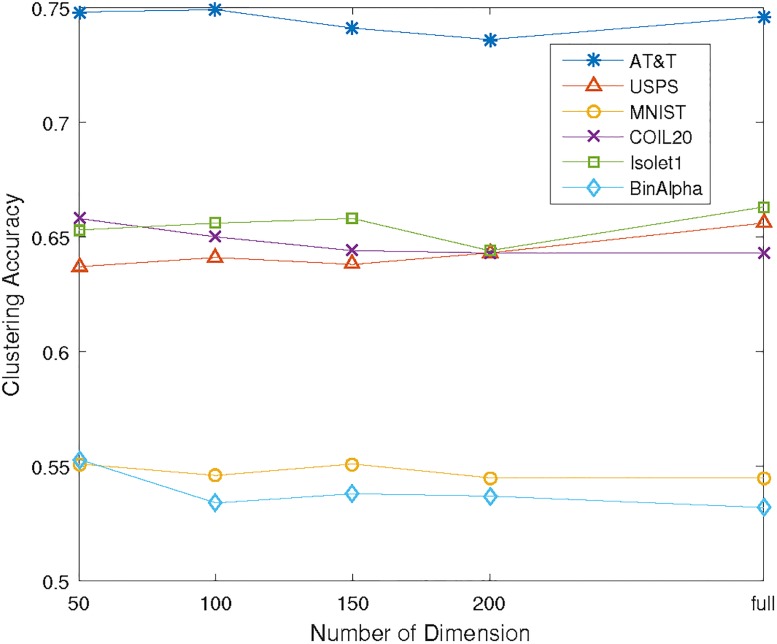
Clustering accuracy of gEKM against the number of dimension of PCA subspace on six datasets. where “full” on the x-axis denotes the full dimension of the datasets as indicated on Table 1.

Table 2 reports the clustering accuracy on the six datasets using K-means, NMF, FCM, Ncut, MinMaxCut, EKM and gEKM. One can see that (1) Ncut and MinMaxCut consistently outperform the existing NMF, FCM and Kmeans methods. (2) The proposed EKM beats Ncut and MinMaxCut in most of the cases. (3) MinMaxCut and Ncut perform nearly to proposed EKM and slightly better in some cases. (4) The integrated gEKM consistently outperforms the other six methods including MinMaxCut and Ncut which verifies the effectiveness of the integration. In order to elaborate the performance of proposed methods, we evaluate the clustering experiments in different PCA subspace with the dimension of *p* = 50, 100, 150, 200 on the six datasets. The obtained clustering accuracies are given in Tables 3 to 6. First of all, the results on different PCA subspaces are consistent as the full space as we discussed above. Furthermore, as shown in Fig 6, the clustering results on PCA subspace can achieve competitive or even slight better than the full space, which implies the effectiveness of subspace discovery.

**Table 2:** Clustering accuracy of six datasets in full dimension.

Dataset	Kmeans	FCM	NMF	Ncut	MinMaxCut	EKM	gEKM
AT&T	0.715	0.701	0.721	0.731	0.730	**0.733**	**0.746**
USPS	0.618	0.626	0.624	0.644	**0.646**	0.621	**0.656**
MNIST	0.494	0.501	0.518	0.531	0.532	**0.534**	**0.545**
COIL20	0.625	0.615	0.631	**0.638**	0.635	0.634	**0.643**
Isolet1	0.626	0.624	0.628	0.651	0.645	**0.654**	**0.663**
BinAlph	0.495	0.511	0.502	0.511	**0.514**	0.512	**0.532**

**Table 3:** Clustering accuracy of six datasets in PCA subspace when p = 50.

Dataset	Kmeans	FCM	NMF	Ncut	MinMaxCut	EKM	gEKM
AT&T	0.713	0.713	0.715	**0.736**	0.731	0.734	**0.748**
USPS	0.604	0.619	0.621	0.627	0.632	**0.636**	**0.637**
MNIST	0.521	0.523	0.524	**0.545**	0.537	0.536	**0.551**
COIL20	0.620	0.631	0.627	0.633	0.640	**0.652**	**0.658**
Isolet1	0.626	0.627	0.626	0.641	0.635	**0.646**	**0.653**
BinAlph	0.498	0.510	0.518	0.534	0.533	**0.541**	**0.553**

**Table 4:** Clustering accuracy of six datasets in PCA subspace when p = 100.

Dataset	Kmeans	FCM	NMF	Ncut	MinMaxCut	EKM	gEKM
AT&T	0.711	0.701	0.711	0.737	0.731	**0.738**	**0.749**
USPS	0.608	0.625	0.625	0.632	0.622	**0.635**	**0.641**
MNIST	0.516	0.521	0.525	0.534	0.535	**0.539**	**0.546**
COIL20	0.618	0.625	0.630	0.633	0.634	**0.637**	**0.650**
Isolet1	0.628	0.623	0.627	0.637	0.639	**0.645**	**0.656**
BinAlph	0.481	0.515	0.518	0.523	0.522	**0.528**	**0.534**

**Table 5:** Clustering accuracy of six datasets in PCA subspace when p = 150.

Dataset	Kmeans	FCM	NMF	Ncut	MinMaxCut	EKM	gEKM
AT&T	0.717	0.702	0.702	**0.735**	0.720	0.722	**0.741**
USPS	0.605	0.612	0.627	0.631	0.623	**0.637**	**0.638**
MNIST	0.519	0.511	0.528	0.537	0.539	**0.541**	**0.551**
COIL20	0.607	0.622	0.629	0.631	0.633	**0.638**	**0.644**
Isolet1	0.627	0.622	0.631	0.642	0.640	**0.645**	**0.658**
BinAlph	0.485	0.512	0.519	0.526	0.531	**0.536**	**0.538**

**Table 6:** Clustering accuracy of six datasets in PCA subspace when p = 200.

Dataset	Kmeans	FCM	NMF	Ncut	MinMaxCut	EKM	gEKM
AT&T	0.712	0.708	0.702	**0.731**	0.725	0.723	**0.736**
USPS	0.610	0.616	0.625	0.629	0.631	**0.641**	**0.643**
MNIST	0.512	0.503	0.518	0.525	0.532	**0.539**	**0.545**
COIL20	0.623	0.611	0.621	0.626	0.630	**0.636**	**0.643**
Isolet1	0.619	0.624	0.625	0.631	0.629	**0.633**	**0.644**
BinAlph	0.477	0.512	0.515	0.522	0.525	**0.528**	**0.537**

## Conclusion

In this paper, we firstly propose a Elastic K-means(EKM) framework which provides a elastic clustering solution and prove that the correctness and the convergency of the updating algorithm. Secondly, an integrated framework specified by the combination of EKM and Normalized Cut (gEKM) is proposed to take into account of both attribute data and pairwise relations and the choice of combination parameter is particularly analysed. The experimental results on six benchmark datasets demonstrate the proposed EKM leads to the better clustering accuracy than K-means as well as previous NMF-based algorithms and the popular Fuzzy C-means and Fuzzy K-means. The gEKM has been also been tested to have satisfied performance on data clustering.

## Supporting information

S1 File Supplementary materials. (PDF)

S2 File Supplementary materials.tex file. (TEX)
