# Supplementary material for: Elastic K-means using posterior probability
Source: PLoS One. 2017 Dec 14;12(12):e0188252. doi: 10.1371/journal.pone.0188252 (PMC5730165; doi:10.1371/journal.pone.0188252)
Supplement: S1 File — (PDF) [file pone.0188252.s001.pdf]

# Supplementary Materials

In this supplementary materials, we first prove a useful matrix inequality and then the convergence of Algorithm 1.

## Important Matrix Inequalities

To prove Algorithm 1, we need the following matrix inequalities: (A) Inequality involving quadratic form of matrix $G$ among 2 other matrices where we used the inequality [ [1]]

$$Tr[G^T PGQ] \leq \sum_{ik} (PG^{'}Q)_{ik} \frac{G_{ik}^2}{G_{ik}^{'}} \tag{S1}$$

where $P, Q \in \Re_+^{K\times K}$ , and $P = P^T, Q = Q^T$. $G$ ,$G^{'} \in \Re_+^{n\times K}$. The inequality was proved in [ [1]].

Another useful inequality involving quartic form of matrix $H$ and 2 other matrices

$$\begin{aligned} &Tr(HAH^T HBH^T) \\ &\leq \sum_{i=1}^{n}\sum_{k=1}^{K}(\frac{H^{'}AH^{'T}H^{'}B + H'BH'THA}{2})_{ik}\frac{H_{ik}^4}{H_{ik}^{'3}} \end{aligned} \tag{S2}$$

where $A, B \in \Re_+^{K\times K}$ ,$H$ ,$H^{'} \in \Re_+^{n\times K}$ are nonnegative matrices; and $A, B$ are symmetric: $A = A^T, B = B^T$.

A slight variant of the above is

$$\begin{aligned} &Tr(H^T AHH^T BH) \\ &\leq \sum_{i=1}^{n}\sum_{k=1}^{K}(\frac{AH^{'}H^{'T}BH^{'} + BH^{'}H^{'T}AH^{'}}{2})_{ik}\frac{H_{ik}^4}{H_{ik}^{'3}} \end{aligned} \tag{S3}$$

where $A, B \in \Re_+^{n\times n}$.

This inequality is useful when analyzing objective functions involving 4-th order matrix polynomials, such as the NMF of Eq.(1). We will use this inequality to prove the convergence of the algorithm of Eq.(S2).

**Proof of the inequality Eq.(S3)**

Let $H_{ik} = H_{ik}^{'}\mu_{ik}$. The 2nd term in RHS of Eq.(S3) is

$$\sum_{ik}(H^{'}BH^{'T}H^{'}A)_{ik}\frac{H_{ik}^4}{H_{ik}^{'3}} = \sum_{ijkrpq} H_{ip}^{'}B_{pq}H_{jq}^{'}H_{jr}^{'}A_{rk}H_{ik}^{'}\mu_{ik}^4 \tag{S4}$$

Now, switching indexes: $i \Longleftrightarrow j$, $p \Longleftrightarrow q$, $r \Longleftrightarrow k$, we obtain

$$\sum_{ik}(H^{'}BH^{'T}H^{'}A)_{ik}\frac{H_{ik}^4}{H_{ik}^{'3}} = \sum_{ijkrpq} H_{jq}^{'}B_{qp}H_{ip}^{'}H_{ik}^{'}A_{kr}H_{jr}^{'}\mu_{jr}^4 \tag{S5}$$

The 1st term in RHS of Eq.( S3 ) is

$$\sum_{ik}(H^{'}AH^{'T}H^{'}B)_{ik}\frac{H_{ik}^4}{H_{ik}^{'3}} = \sum_{ijkrpq} H_{jr}^{'}A_{rk}H_{ik}^{'}H_{ip}^{'}B_{pq}H_{jq}^{'}\mu_{jq}^4 \tag{S6}$$

Now, switching indexes: $i \Longleftrightarrow j$,$p \Longleftrightarrow q$,$r \Longleftrightarrow k$, we obtain

$$\sum_{ik}(H^{'}AH^{'T}H^{'}B)_{ik}\frac{H_{ik}^4}{H_{ik}^{'3}} = \sum_{ijkrpq} H_{ik}^{'}A_{kr}H_{jr}^{'}H_{jq}^{'}B_{qp}H_{ip}^{'}\mu_{ip}^4 \tag{S7}$$

Carefully examination of the RHS of Eqs.( S4 - S7 ) show that they are identical except $\mu^4$ terms. Adding Eqs.( S4 - S7 ), we obtain that the RHS of Eq.( S3 ) is equal to

$$\sum_{ijkrpq} H_{ip}^{'}B_{pq}H_{jq}^{'}H_{jr}^{'}A_{rk}H_{ik}^{'}\frac{\mu_{ik}^4+\mu_{jr}^4+\mu_{jq}^4+\mu_{ip}^4}{4} \tag{S8}$$

The LHS of Eq.( S3 ) is equal to

$$\sum_{ijkrpq} H_{ip}^{'}B_{pq}H_{jq}^{'}H_{jr}^{'}A_{rk}H_{ik}^{'}\mu_{ik}\mu_{jr}\mu_{jq}\mu_{ip} \tag{S9}$$

Therefore, if we can establish

$$\mu_{ik}\mu_{jr}\mu_{jq}\mu_{ip} \leq \frac{\mu_{ik}^4+\mu_{jr}^4+\mu_{jq}^4+\mu_{ip}^4}{4}, \tag{S10}$$

then the inequality Eq.( S3 ) holds. For any $a, b, c, d > 0$, we have

$$\begin{aligned} a^4+b^4 \geq 2a^2b^2, c^4+d^4 \geq 2c^2d^2, \\ (ab)^2+(cd)^2 \geq 2(ab)(cd). \end{aligned} \tag{S11}$$

Thus

$$a^4+b^4+c^4+d^4 \geq 2(a^2b^2+c^2d^2) \geq 4(ab)(cd). \tag{S12}$$

This is Eq.(S10). □

## Convergence Proof of Algorithm 1

**Proof.** We use the auxiliary function approach[15]. Function $Z(G, G^{'})$ is an auxiliary function of $J(G)$ if it satisfies

$$Z(G, G^{'}) \geq J(G), Z(G, G) = J(G). \tag{S13}$$

Once an auxiliary function is found, we define

$$G^{t+1} = \arg\min_{G} Z(G, G^{(t)}) \tag{S14}$$

Then, by construction, we have

$$J(G^{(t)}) = Z(G^{(t)}, G^{(t)}) \leq Z(G^{(t+1)}, G^{(t)}) \leq J(G^{(t+1)}) \tag{S15}$$

This proves that $J(G^{(t)})$ is monotonically decreasing. The key steps in the remainder of the proof are: (1) Finding an appropriate auxiliary function of the objective function; (2) Finding the global maxima of the auxiliary function; (3) Showing that the update rule of Eq.(S4) gives the global minima of Eq.(S14).

**Auxiliary function**

The objective function Eq.(7) is

$$\begin{aligned} Tr[-2G^T(A-B)G+G^T(A-B)GG^TG] = \\ Tr[2G^TBG+G^TAGG^TG]-2G^TAG-G^TBGG^TG] \end{aligned} \tag{S16}$$

where $A = (X^T X)_+ \geq 0$ and $B = (X^T X)_- \geq 0$.

Now, we construct an auxiliary function $Z(G, G^{'})$ as the following

$$Z(G, G^{'}) = RHSofEq.(S18) + RHSofEq.(S20) \\ -RHSofEq.(S21) - RHSofEq.(S22). \tag{S17}$$

This is done by introducing the upper bounds of the first two terms of Eq.(S16) and the lower bounds of the last two terms (ignoring the negative sign).

The upper bounds of the first terms of Eq.(S16) is

$$Tr[G^T BG] \leq \sum_{ik} (BG^{'})_{ik} \frac{G_{ik}^2}{G_{ik}^{'}} \leq \sum_{ik} (BG^{'})_{ik} \frac{G_{ik}^4 + G_{ik}^{'4}}{2G_{ik}^{'3}} \tag{S18}$$

and (derived from $2gg^{'} \leq g^2 + g^{'2}$)

$$G_{ik}^2 \leq \frac{G_{ik}^4 + G_{ik}^{'4}}{2G_{ik}^{'2}} \tag{S19}$$

Clearly, Eq.(S18) becomes equality when $G = G^{'}$, satisfying Eq.(S13).

The upper bound of 2nd term is

$$R \leq \sum_{ik} \frac{(AGG^T G)_{ik} + (GG^T AG)_{ik}}{2} \frac{G_{ik}^4}{G_{ik}^{'3}} \tag{S20}$$

where $R = Tr[G^T AGG^T G]$, Eq.(S20) becomes equality when $G = G^{'}$, satisfying E-q.(S13).

The lower bound of 3rd term is

$$Tr[2G^T BG] \leq 2 \sum_{ijk} G_{ik}^{'} A_{ij} G_{jk}^{'} (1 + \frac{G_{ik} G_{jk}}{G_{ik}^{'} G_{jk}^{'}} \tag{S21}$$

where we use $z \geq 1 + \log z$, $z > 0$ and setting $z = \frac{G_{ik} G_{jk}}{G_{ik}^{'} G_{jk}^{'}}$. Eq.(S21) becomes equality when $G = G^{'}$, satisfying Eq.(S13).

The lower-bound of 4th term is

$$R \leq \sum_{ii^{'}jkl} G_{ik} A_{ij} G_{jl} G_{i^{'}l} G_{i^{'}k} (1 + \frac{G_{ik} G_{jl} G_{i^{'}l} G_{i^{'}k}}{G_{ik}^{'} G_{jl}^{'} G_{i^{'}l}^{'} G_{i^{'}k}^{'}}) \tag{S22}$$

where $R = Tr[G^T AGG^T G]$, Eq.(S22) becomes equality when $G = G^{'}$, satisfying E-q.(S13). This completes the construction of the auxiliary function $Z(G, G^{'})$.

**Finding global minima.**

Now we seek the global minima of $f(G) \equiv Z(G, G^{'})$ while fixing $G^{'}$. The gradient w.r.t. $G$ is [collecting RHS of Eqs.(S18,S20,S21,S22)]

$$\begin{aligned} & \frac{1}{2} \frac{\partial Z(G, G^{'})}{\partial G_{ik}} \\ & = \frac{(BG^{'})_{ik} G_{ik}^3}{G_{ik}^{'3}} + [(AG^{'} G^{'T} G^{'})_{ik} + (G^{'} G^{'T} AG^{'})_{ik}] \frac{G_{ik}^3}{G_{ik}^{'3}} \\ & - \frac{G_{ik}(BG^{'})_{ik}}{G_{ik}} - \frac{(BG^{'} G^{'T} G^{'})_{ik} + (G^{'} G^{'T} BG^{'})_{ik}}{G_{ik}} \end{aligned} \tag{S23}$$

The Hessian (2nd derivative) are

$$\frac{\partial^2 Z(Q, Q^{'})}{\partial G_{ik} \partial G_{jl}} = \delta_{ij} \delta_{kl} D_{ik} \tag{S24}$$

where

$$
\begin{aligned}
&D_{ik} \\
&= 6\frac{(BG^{'})_{ik}G_{ik}^2}{G_{ik}^{'3}} + 6[(AG^{'}G^{'T}G^{'})_{ik} + (G^{'}G^{'T}AG^{'})_{ik}]\frac{G_{ik}^2}{G_{ik}^{'3}} \\
&+ 2\frac{G_{ik}(BG^{'})_{ik}}{G_{ik}^2} + 2\frac{(BG^{'}G^{'T}G^{'})_{ik} + (G^{'}G^{'T}BG^{'})_{ik}}{G_{ik}^2}
\end{aligned}
\tag{S25}
$$

Clearly the Hessian is diagonal with positive elements, and hence is semi-definite positive. Therefore $f(Q) = Z(Q, Q^{'})$ is a convex function. The global minima is obtained by setting the gradient Eq.(S23) to zero. We obtain

$$
G_{ik}^4 = G_{ik}^{'4}\frac{[BG^{'} + AG^{'}G^{'T}G^{'} + G^{'}G^{'T}AG^{'}]_{ik}}{[AG^{'} + BG^{'}G^{'T}G^{'} + G^{'}G^{'T}BG^{'}]_{ik}} \tag{S26}
$$

as the optimal solution to Eq.(S12) (note $G^t \leftarrow G^{'}, G^{t+1} \leftarrow G$). Eq.(S26) which is identical to the update rule Eq.(S4). Thus we proved that under the update rule Eq.(S4) the objective function $J(G)$ monotonically decrease. □

## References

1. Ding C, Li T, Jordan MI. Convex and semi-nonnegative matrix factorizations. IEEE Transactions on Pattern Analysis and Machine Intelligence. 2010;24:45–55.
